# Rapid assessment of myocardial infarct size in rodents using multi-slice inversion recovery late gadolinium enhancement CMR at 9.4T

**DOI:** 10.1186/1532-429X-13-44

**Published:** 2011-09-05

**Authors:** Anthony N Price, King K Cheung, Shiang Y Lim, Derek M Yellon, Derek J Hausenloy, Mark F Lythgoe

**Affiliations:** 1 UCL Centre for Advanced Biomedical Imaging, Department of Medicine and UCL Institute of Child Health, University College London, UK; 2 The Hatter Cardiovascular Institute, University College London Hospital and Medical School, London, UK

## Abstract

**Background:**

Myocardial infarction (MI) can be readily assessed using late gadolinium enhancement (LGE) cardiovascular magnetic resonance (CMR). Inversion recovery (IR) sequences provide the highest contrast between enhanced infarct areas and healthy myocardium. Applying such methods to small animals is challenging due to rapid respiratory and cardiac rates relative to *T*_1 _relaxation.

**Methods:**

Here we present a fast and robust protocol for assessing LGE in small animals using a multi-slice IR gradient echo sequence for efficient assessment of LGE. An additional Look-Locker sequence was used to assess the optimum inversion point on an individual basis and to determine most appropriate gating points for both rat and mouse. The technique was applied to two preclinical scenarios: i) an acute (2 hour) reperfused model of MI in rats and ii) mice 2 days following non-reperfused MI.

**Results:**

LGE images from all animals revealed clear areas of enhancement allowing for easy volume segmentation. Typical inversion times required to null healthy myocardium in rats were between 300-450 ms equivalent to 2-3 R-waves and ~330 ms in mice, typically 3 R-waves following inversion. Data from rats was also validated against triphenyltetrazolium chloride staining and revealed close agreement for infarct size.

**Conclusion:**

The LGE protocol presented provides a reliable method for acquiring images of high contrast and quality without excessive scan times, enabling higher throughput in experimental studies requiring reliable assessment of MI.

## Background

Cardiovascular magnetic resonance (CMR) has emerged as a valuable tool in the field of small animal cardiovascular research [1-6]. However, many of the CMR techniques that are now used as standard in the clinical setting remain a significant challenge to implement in small animals especially at high field [6], where relatively few optimised protocols are available as standard on experimental systems. One such technique, regarded as the gold standard for assessing myocardial infarction (MI) in patients [7], is late gadolinium enhancement (LGE) CMR. The extent of the LGE region is typically assessed using an inversion recovery (IR) breath-hold sequence, which can be readily applied to larger animals used in cardiovascular research [8,9]. When it comes to assessing LGE in small animals, such as rat and mouse, these methods become difficult to implement due to the rapid heart and respiratory rates. This is confounded by the fact most CMR of small animals is typically done at high field where the inherent *T*_1 _relaxation of myocardium is substantially longer than at clinical field strengths.

For these reasons the majority of reported implementations of LGE CMR in small animals have tended to use more conventional *T*_1_-weighted sequences, namely short repetition time (TR), high flip angle, cine gradient echo [10,11]. Nevertheless, there have been a limited number of studies recently that have used IR sequences to assess LGE at high field in both mice [12-14] and rats [15]. In order to achieve the maximum contrast between infarcted myocardium (where extracellular contrast agents such as Gd-DTPA will accumulate to enhance MR signal) and the normal healthy myocardium, it is necessary to use an IR technique with the appropriate inversion time (TI). Finding the optimum TI, in order to sufficiently null the signal from normal myocardium, can be difficult to assess at high field, as TI is typically several times longer than the R-R wave interval, even after relatively high doses of contrast agent. Thus achieving the best contrast whilst also maintaining high quality images that have optimum gating, relative to both the respiratory and cardiac cycles, is key to accurate assessment of MI using LGE CMR in small animals.

In recent reported implementations of IR sequences to assess LGE in mice at 9.4T [12,13], 7T [16] and rats at 4.7T [15] the approach has been to use a segmented spoiled gradient echo (GRE) or FLASH sequence, where a number of echoes or lines (with typical echo-train lengths of 4-16 being reported) are acquired following each inversion pulse. However, the alternative approach for 2D imaging is to use the relatively small gating window available to acquire a multi-slice GRE acquisition.

Here we present a robust yet fast protocol, and gating strategy, for assessing MI in rodents based on a combination of Look-Locker (LL) [17-19] and multi-slice IR-GRE sequences. We have employed a LL technique to evaluate the optimum null point to provide good tissue contrast and multi-slice IR-GRE to produce high quality images of enhanced regions following administration of Gd-DTPA. The area of infarction measured *in vivo *by CMR is compared to *ex vivo *triphenyltetrazolium chloride (TTC) staining - an established technique to assess viable myocardium - to evaluate the correlation between the two methods. The protocol was applied to two different practices used by cardiovascular MI researchers i) rats in the acute phase of a reperfused MI and ii) mice 2 days after permanent ischemic MI in order to assess a broad range of methodologies applied to small animal models of myocardial infarction.

## Methods

### Rat model of acute myocardial infarction

All animal procedures were approved by the University College London Ethics Committee and the Home Office (London, UK), and conducted under the Animal (Scientific Procedures) Act 1986. Wistar rats (9 males, aged 7 - 8 weeks) were anaesthetised with sodium thiopentone (i.p., 120 mg/kg, Intraval, U.K.) and underwent surgery to induce myocardial infarction. Briefly, an endotracheal tube was inserted via a tracheotomy for mechanical ventilation, after which a small thoracotomy was performed along the 4th lateral intercostal space to expose the heart. The left anterior descending (LAD) coronary artery was occluded close to its origin with a snare occluder for 30 minutes, which was then released for reperfusion. Following visual verification of successful reperfusion (i.e. disappearance of pallor and return of blood flow), the chest was closed, and spontaneous breathing was re-established. Animals were placed in the MR scanner and post-MI images were acquired at 2 hours from reperfusion.

### Mouse model of myocardial infarction

B6Sv129 mice (6 males, 9-11 weeks old) were anesthetised by an intraperitoneal injection of ketamine (75 mg/kg) and medetomidine hydrochloride (1 mg/kg). The trachea was exposed through a mid-line incision and intubated through the oral pharynx for artificial respiration. Through a left anterior thoracotomy, the heart was exposed with aid of a mini-retractor. Ligation of the LAD artery was performed at a level 1 mm below the edge of the left atrium. Successful LAD occlusion was confirmed by a sudden discoloration of the anterior wall of the left ventricle. After the coronary intervention, the chest wall and skin were closed, animals were given atipamezole hydrochloride (5 mg/kg, i.p.) as a sedation reversal agent and buprenorphine (0.1 mg/kg, intramuscular) as an analgesic. After spontaneous respiration had resumed, the animal was weaned from the respirator and allowed to recover on a heating pad. Mice were imaged at 2 days post-surgery.

### CMR

Anaesthetised animals were placed onto an animal cradle with a water-heating system to maintain body temperature. Oxygen and isoflurane (1-2%) was provided via a nose cone and a scavenging system was used to remove anaesthetic gases. A neonatal apnoea sensor was placed on the abdomen for respiratory gating and electrocardiogram (ECG) was obtained by needle electrodes inserted subcutaneously. Cardio-respiratory monitoring and gating were performed using an MR-compatible system (SA Instruments, NY).

Imaging was performed using a 9.4T VNMRS horizontal bore scanner (Varian Inc. Palo Alto, CA) with a shielded gradient system (400 mT/m). For rats the RF excitation was achieved using a 72 mm de-tuneable volume coil and signal received using a 4-element array cardiac coil (Rapid Biomedical GmbH, Germany). Mice were imaged using a 33 mm quadrature volume coil (Rapid Biomedical GmbH, Germany) and 1000 mT/m gradient insert. Cardiac images of conventional orientations (2-chamber, 4-chamber and short-axis) were obtained as described below. Initially, axial and longitudinal scout images were acquired to plan the 2-chamber long-axis view. A 4-chamber long-axis view was then acquired perpendicular to the 2-chamber orientation, after which a series of short-axis images was obtained perpendicular to the LV long-axes from the 2- and 4-chamber images - in a similar fashion described in [20]. In order to cover the whole LV from apex to base, typically 15 short-axis image slices were acquired for rats and 10 for mice.

### Cine sequence

A double (respiratory + cardiac) gated spoiled gradient echo sequence was used to acquire cine cardiac images, and used to calculate left ventricular mass (LVM). Imaging parameters for rats were as follows; echo time (TE) = 1.7 ms, repetition time (TR) = ~7.5 ms, flip angle = 15°, slice thickness = 1 mm, field of view (FOV) = 40 × 40 mm, matrix size = 192 × 192, number of signal averages = 1. Twenty time-frames were recorded for every cardiac cycle, and phase encoding was incremented linearly following each cycle. A single short-axis slice was obtained in approximately 45 seconds, leading to a total scan time for cine data of around 10 to 15 minutes. In mice the following parameters were adjusted: TE = 1.1 ms, TR = 4-5 ms, FOV = 25.6 × 25.6 mm, matrix size = 128 × 128, with 10 slices acquired taking ~5 minutes.

For delayed contrast enhancement 0.6 mmol/kg Gd-DTPA (Magnevist, Schering AG, Germany) was injected, via an intra-venous route for rats (tail vein), following initial baseline scanning. In mice contrast agent was administered via an intra-peritoneal route, due to the difficulty and unreliability in cannulating the mouse tail vein.

### Look-Locker (LL) TI evaluation

**Figure 1:**
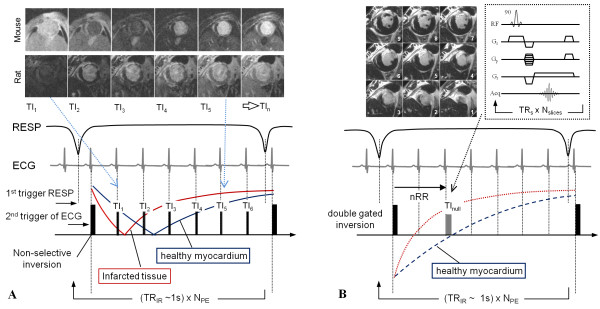
**Pulse sequence diagrams for LGE infarct imaging**. a) Look-Locker pulse sequence and gating strategy used to acquire multiple TI images following adiabatic inversion, images from TI_1_-TI_6 _time points are shown for both mouse and rat, where the indices relate to number of R-wave triggers since the double gated inversion pulse. b) LGE IR sequence diagram follows the same gating and acquisition scheme but acquires a full flip angle multi-slice GRE stack at the nearest TI value to null healthy myocardium. In addition, TR_s _is time between slice acquisition pulses, TR_ir _is time between inversion pulses, N_PE _is number of phase encodes, and N_slices _is number of slices. Also note that as timings between TI frames are dependent on ECG gating they differ between rat and mouse (i.e on average RR of rat = 151 ms, RR of mouse = 111 ms).

Following contrast agent administration and short time delay of ~10 minutes to allow for late enhancement to establish a Look-Locker inversion recovery sequence (Figure 1a) was used to acquire multiple TI images following a double gated non-selective adiabatic inversion pulse. Typically 6-8 frames, with corresponding TI range ~100-900 ms, were acquired, consisting of a single selected slice containing both MI and healthy myocardium at each TI point. All TI frames were ECG gated, ensuring images are collected from the same part of the cardiac cycle, but as a result restricting TI points to multiples of the R-R period. An optional QRS delay can be included to shift images from end-diastolic to end-systolic views. Additional parameters for rats: FOV = 40 × 40 mm, matrix size = 192 × 192, slice thickness = 1 mm, TE = 1.7 ms, TR_ir _= ~1 s (depending upon respiratory rate), flip angle = 10°, number of signal averages = 1, total acquisition time ~3 minutes. The following parameters only were adjusted in mice to also provide 200 μm in-plane resolution (FOV = 25.6 × 25.6 mm, matrix = 128 × 128), and TE = 1.1 ms.

### LGE acquisition

The LL scan was followed by a high SNR multi-slice IR-GRE sequence with single TI point and flip angle of 90°; maintaining other parameters was important to achieve a similar effective nulling of healthy myocardium compared to LL acquisition. TI is selected from the initial multiple frames of the LL scan to be the closest gated TI point which most effectively nulls healthy myocardium and consequently provides the best contrast whilst also maintaining image quality from the optimum gating strategy. The repetition time between each slice pulse (TR_s_) was 3.6 ms, with a sequential acquisition order starting at the apex; typically 9 slices were needed to cover the area of infarction in the rat. Mice were also imaged using the same LGE protocol as described above except again for the following parameters; TE = 1.1 ms, TR_s _= 3.1 ms, FOV = 25.6 ×25.6 mm, matrix size = 128 × 128, and 7 slices typically needed to cover area of infarction.

### Ex vivo infarct measurement by TTC staining

After all imaging was completed, rat hearts were extracted for infarct quantification by triphenyltetrazolium chloride (TTC) staining and planimetry. Hearts were extracted and frozen at -20°C for 2 hours and cut into 2 mm-thick slices. To demarcate infarcted myocardium from viable myocardium, heart slices were put into 1% TTC solution at 37°C for 15 minutes. Heart slices were then fixed in 4% PFA overnight, and placed between two Perspex sheets to be imaged using a colour flat-bed scanner at 600 dpi resolution. Images were subsequently analysed using a semi-automatic, colour-based segmentation technique in ImageJ software (NIH).

### Analysis

Obtaining meaningful estimates of background noise in images acquired using receive array coils is difficult to achieve without having acquired additional noise reference scans or repeated acquisitions [21]. For this reason we decided not to quote SNR/CNR values and instead opted for the contrast resolution to give a better comparative estimate of contrast between the relevant tissues. Contrast resolution is defined as CR = (S_A_-S_B_)/(S_A_+S_B_). Signal intensities in the LV blood pool, healthy and infarcted myocardium were measured from 3 consecutive slices containing both infarcted and healthy myocardium using ImageJ. For each slice, an elliptical region of interest (ROI) was selected to include the largest continuous area of enhanced myocardium possible; a second ROI of the same size was then placed diagonally opposite for measurement of remote healthy myocardium, typically within the septum.

Left ventricular mass was assessed from the full stack of short-axis cine images using freely available software Segment http://segment.heiberg.se/[22] by means of the semi-automated LV tracking tool and valve plane tracked on the 2 and 4-chamber views as described in [23]. Infarct volume was calculated from the late gadolinium images in Segment using the auto delineate (weighted method) function which is constrained by the endo and epi-cardial borders defined in the prior segmentation of LVM. Infarct volume is expressed as percentage of left ventricular mass for TTC comparison. Infarct volumes by *ex vivo *TTC and *in vivo *CMR were blinded and anonymised prior to analysis. Bland-Altman and linear regression analysis was applied to the LGE-CMR and TTC staining data. All statistical analysis was performed in GraphPad Prism (v4.01 for Windows).

## Results

**Figure 2:**
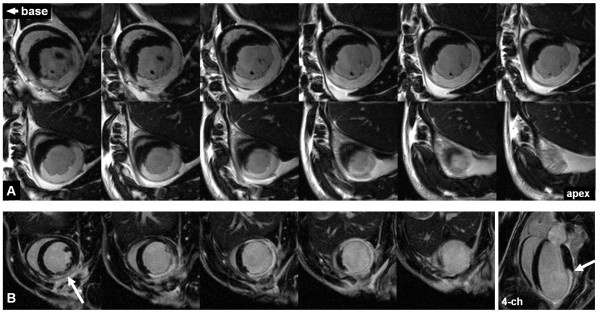
**IR-GRE multi-slice LGE images of rat and mouse**. Example images of late gadolinium enhancement in an acute reperfused rat MI model using the inversion recovery sequence described. 12 contiguous slices are shown (a), from just above mid-ventricle down through to apex, highlighting the infarcted region. LGE images from a mouse (b) 2 days following permanent occlusion of LAD, an additional separately acquired slice taken in the long axis 4-chamber view is shown to illustrate extent of infarction (arrows).

All animals that received surgery were successfully imaged and revealed enhancement in LGE images. The LL sequence described and illustrated in Figure 1a was applied at a time point approximately 10 minutes after contrast administration, followed immediately by a full short-axis multi-slice acquisition (Figure 1b) using the most appropriate gated TI point selected from LL images. Subsequently, the full IR LGE image stacks (Figure 2) was acquired at an average time point of 13 ± 3 (rats) and 14 ± 3 (mice) minutes following injection of contrast. Due to the nature of the gating strategy it is beneficial that respiratory and cardiac rates remain steady to ensure consistent image quality and contrast. The respiratory cycle, typically maintained to ~60 breaths per minute by small adjustments to anaesthesia, dictates the effective TR_ir _and ultimately the contrast and effective signal null points. There is a trade-off between acquisition speed and allowing for sufficient recovery of magnetisation. However, in this approach we opted for a faster acquisition where the condition of TR_ir_>>*T*_1 _is not fully satisfied, as we were not aiming to measure *T*_1 _directly but to obtain images of sufficient SNR and quality to measure infarct volume. The dose of contrast agent and parameters used in this study resulted in a typical TI point being between 2 to 3 R-wave intervals following inversion (300-450 ms) in rats. In mice this was typically 3 R-wave intervals (in 9 out of 11 LGE image sets from the 6 animals) leading to an average TI value of ~330 ms. The average heart rate was recorded as 397 ± 9 (rats) and 540 ± 64 (mice) bpm during acquisition of both LL and LGE images.

**Figure 3:**
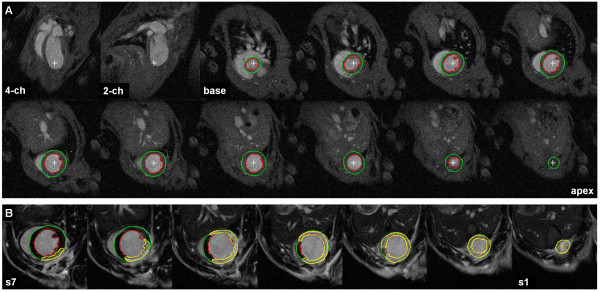
**Segmentation of LV myocardium and infarcted tissue**. Example segmentation of a mouse heart; endo and epi-cardial borders are initially defined in order to calculate LVM and all necessary functional parameters from the cine data, shown in (a) on the end-diastolic frame. The LV myocardial boundaries are then also used to aid segmentation of infarct area using the short axis LGE images (b).

Example LGE images of a rat from a full short-axis stack are illustrated in Figure 2a with twelve slices shown from apex up towards the base of the rat heart. The images are acquired here at end-systolic phase of the cardiac cycle. The same technique applied to mice 48 hours following surgery also achieved high contrast and image quality as seen in Figure 2b. The MI model used for mice employed permanent occlusion of the LAD and different route of administration for contrast agent (i.p.) but the area of infarct can still clearly be discriminated without adjustments in the time allowed between injection and LGE imaging. The mouse images are acquired here at end-diastolic phase with acquisition immediately following R-wave trigger. Image quality tended to be slightly more consistent when acquisition was gated in the end-diastolic phase of the cardiac cycle. This is likely a result of small variations in the heart rate over the total image acquisition period. Here an additional long-axis 4-chamber LGE image has been acquired which can be used to aid segmentation of the infarcted region around the apex which can be more difficult to assess in mice using 2D methods, especially with more chronic (thinner) infarcts. However, using the LV segmentation from the stack of cine images, which provides the measure of LVM, can also assist segmentation of the LGE region, as shown in Figure 3.

The LGE slices are acquired sequentially up from the apex, with a TR_s _= 3.1 ms and 7 slices typically needed, the mouse LGE region can be covered within the time frame of 22 ms frame the R wave trigger. Therefore the majority of the LGE region is acquired close enough to the end diastolic cine frame, with just the upper slices starting to edge into systole. Furthermore, as it is the endo and epi-cardial borders surrounding the LGE regions that are being utilised, these are inevitably akinetic and adjusting these boundaries or taking later cine frame segmentations to fit the later LGE slices is rarely necessary.

**Figure 4:**
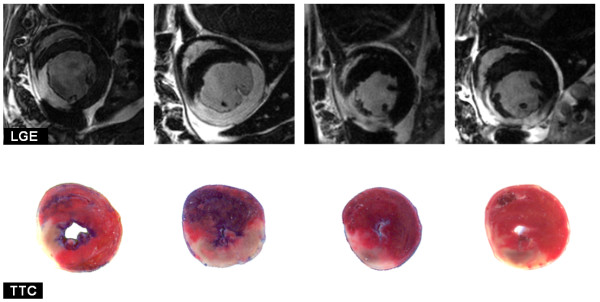
**LGE example images with corresponding *ex vivo *TTC staining**. Selected LGE images and corresponding TTC sections from four animals (rats 2 hours post reperfused MI). LGE and TTC agree closely through the range of infarct sizes shown. TTC stains viable myocardium red leaving the infarct area white. In addition, the first 3 hearts were also stained for perfusion with Evans blue prior to TTC.

**Figure 5:**
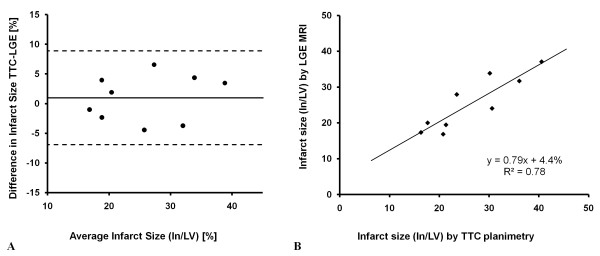
**Bland-Altman and linear regression plots of infarct size measured by LGE CMR and TTC staining**. Bland-Altman (a) and linear regression plots (b) showing the difference and correlation in infarct size, as a percentage of LV, measured by LGE-CMR and TTC planimetry.

Figure 4 shows four example *in vivo *short axis LGE images from rats alongside the corresponding *ex vivo *TTC staining revealing close correlation between the two techniques. The infarct size measurements (In/LV) for all rats is presented in Figure 5 using a Bland-Altman plot revealing that no significant bias was observed between LGE CMR and TTC staining (1.0 ± 3.9%, mean ± SD). Linear regression analysis also reveals close agreement between the two methods with y = 0.8 × + 4.4 and R^2 ^= 0.78. Unfortunately, it was not possible to perform the TTC staining in mice due to those animals being part of another long term study.

Contrast resolution between infarcted and remote myocardium was measured in LGE images as 82.6 ± 2.6% in rats and 73.9 ± 13.1% in mice; sufficient for the purpose of infarct volume segmentation and measurement. Contrast resolution between infarct and blood pool was 27.1 ± 3.8% in rats and -1.4 ± 7.8% in mice, which illustrates why LV segmentation from cine data was particularly useful in mice.

## Discussion

CMR provides a remarkably clear and non-invasive way to assess the extent of myocardial infarction with the use of extracellular contrast agents. It is undoubtedly the gold standard way to assess the extent and location of infarcted tissue in patients, with a typical LGE imaging protocol based around an inversion recovery sequence. However, when it comes to imaging small animals at high field strengths there are varying opinions on the best methods to use. In all the recent implementations of IR sequences to assess LGE in mice at 9.4T [12,13], 7T [16] and rats at 4.7T [15] the approach has been to use a segmented FLASH sequence, where a number of echoes/lines (ETL = 4-16) are acquired following each inversion pulse, with some variations in gating strategy. In the work described by Chapon et al. [13], LGE was assessed in a permanent occluded mouse model of MI using segmented IR-FLASH. ECG gating was applied to the inversion pulse and data acquired at a set TI, and subsequently repeated for many TI values to evaluate the best value for optimum contrast. Bohl et al. [12] used a segmented 3D IR-GRE sequence to achieve high resolution scanning of MI in mice at 9.4T. This method is likely to be the best approach to use when high resolution images are necessary, particularly as achieving high through plane resolution would generally limit the use of conventional 2D acquisition approaches. The later study also employed IR-(snapshot)FLASH to map *T*_1 _of infarct and normal myocardium temporally following both intravenous and intraperitoneal administration of Gd-DTPA(-BMA).

In a study by Thomas et al. [15] IR sequences were compared directly to *T_1 _*weighted cine in rats at 4.7T and concluded that *T*_1_-weighted cine was preferred due to the speed advantage of acquiring both global cardiac function and LGE data in one. This study utilised a necrotic-specific contrast agent removing the problem of dynamic changes in contrast that are exhibited by standard Gd-chelated agents. In a similar study by Protti et al. [16] a Gd-DTPA bolus was given i.p. in mice at 7T and cine-FLASH images acquired in the 20-30 minute window compared to IR-FLASH performed in the 30-50 minute period following injection. Cine-FLASH was also preferred here to a segmented IR for being less user dependent while providing similar accuracy. A likewise comparison of *T_1 _*weighted cine and IR-FLASH at 9.4T, with an additional comparison to a multi-slice IR method as proposed in this study which should offer higher SNR efficiency would also be desirable. The main disadvantage with opting for a single cine scan to assess both LGE and functional parameters is that signal from healthy myocardium needs to be further saturated in order to boost contrast relative to infarcted tissue, which could compromise functional assessment particularly when attempting to delineate the epi-cardial border for measurement of LV mass.

In this study we present an IR-based method for assessing LGE where the acquisition of multiple slices, rather than multiple echoes or phase-encode lines, also yields encouraging results. Although the amount of data acquired at each time point is similar in both methods, acquiring all slices rather than multiple k-space lines allows for the use of higher flip angles and thus higher SNR efficiency can be achieved over the segmented FLASH technique. Acquiring one line of k-space from each slice per inversion allows for the full recovered longitudinal magnetisation to be sampled, whereas segmented FLASH requires lower flip angles to limit the saturation effects through the echo train.

In the presented method here both LL and IR-GRE scans are performed without allowing for complete *T*_1 _recovery, where the overall TR is determined by the respiratory rate. The effect of not allowing for full recovery of longitudinal magnetisation is to reduce the effective null points and signal intensity, which in turn reduces CNR slightly [24]. However, it has been shown that sufficient SNR and contrast can be easily achieved with only single averaged scans employing TR_ir _~ 1s and subsequently TAs ~ 3 minutes.

In this implementation a double gated inversion is used followed by a delay of an integer number of R-wave intervals to ensure all data contributing to an image is acquired at exactly the same point in the cardiac cycle. This approach means that TI is set to a multiple of the R-R wave interval possibly leading to a small deviation from the maximum obtainable contrast, although adding a trigger delay to move to end-systole for example could improve this if desired. The benefits of a more precise and consistent gated acquisition is evident from the clarity of the images, whilst still maintaining sufficiently high levels of contrast between infarcted and viable myocardium. Another advantage of using a multi-slice over segmented FLASH is that each image consists of data acquired from a narrower window in the cardiac cycle. As data contributing to each slice is acquired from a 1 × TR window rather than ETLxTR cardiac motion is reduced.

The speed of acquisition, in addition to the time after injection, is an important factor for LGE using Gd-DTPA, as previous studies have reported relatively rapid pharmacokinetic changes in contrast at the periphery of infarcts [25,26]. The delay of ~10 minutes following injection to LGE imaging was primarily chosen to allow for development of enhancement in the infarcted tissue, but also it has been shown that imaging too soon after administration can lead to an over-estimation of infarct size [25]. However, this delay could be further increased to improve contrast between infarct and remote myocardium when contrast is being administered via the i.p. route. Bohls et al. [12] recently demonstrated maximum *T_1 _*contrast is achieved between 13.5-22 minutes after i.v. injection and 30-40 mins after i.p. injection in mice using a similar concentration and agent (0.5 mmol/Kg Gd-DTPA-BMA). Therefore, although our time-point for rats i.v. is likely close to optimum for maximum contrast, allowing a longer delay for mice after i.p. injections should yield further increases in contrast if scan time constraints allowed. A further potential benefit could be that the contrast between infarct and the blood pool may increase sufficiently to potentially allow for segmentation of infarct area without using the boundaries found from the LV cine segmentation, as was the case for the rat LGE images in this study. However, the recovery of blood *T_1_*, which would improve contrast between blood and infarcted tissue, is confounded by the fact the blood pool signal level is also influenced by inflow enhancement. Therefore, it is hard to suppress the blood signal without also introducing more complicated preparation pulses and timings.

A possible limitation to the study protocol applied here to mice is that it could be argued that resolution should be increased relative to the size of the animal. The biggest benefit would likely come from an increase in the through plane resolution, by decreasing slice thickness and increasing the number of slices. Reducing slice thickness to 0.5 mm for example would be achievable with the gradient capability of the system mentioned, but the resultant slice pack would extend to approximately 1/3 rd of the cardiac cycle, unless the acquisition were split into two interleaved acquisitions. Subsequently this would double scan time and reduce SNR by 50%, however, with this protocol there should still be sufficient levels of signal and contrast to segment LGE area without needing additional signal averages.

The additional step presented in this protocol to optimise the best gated TI point on an individual animal basis using a Look-Locker scan, is useful to confirm arrival and degree of local tissue contrast. However, it is not essential as it has been shown that with the given concentrations of contrast agent used here and at the LGE imaging window following injection we opted for the TI is consistently in the range 300-450 ms, leading to a R-R gate of 2-3 in rats (i.v.) and predominately 3 (occasionally 4) R-waves in mice (i.p.). Both LL and LGE scans are readily acquired in ~3 minutes each thus allowing for a rapid assessment of LGE whilst minimal contrast agent washout occurs. The functional cine assessment, typically desired for most CMR studies of MI, could easily be performed in the time between injection of contrast and LGE imaging to further improve throughput with an additional SNR boost to the cine data that could aid segmentation of LVM. Therefore, a complete functional and LGE assessment could be achieved in around 10-15 minutes from injection of contrast with the 200 μm in-plane resolution used here.

## Conclusion

This study presents a fast and robust protocol for the reliable assessment of myocardial infarction in small animals, using a multi-slice IR sequence at 9.4T. By employing the use of a Look-Locker sequence the optimum inversion time can be checked on an individual animal basis, while the approach used for gating and acquisition provides LGE images of ample contrast and image quality but with scan times of less than 5 minutes. The method has been validated against tissue viability staining in rats following acute reperfused MI, and also shown to work well in mice following non-reperfused MI post 48 hours.

We believe this approach to assessing LGE in small animals offers an accurate and reproducible method to determine infarct size without the need for lengthy scans.

## Competing interests

The authors declare that they have no competing interests.

## Authors' contributions

AP designed and implemented the sequences on the scanner, performed in vivo measurements, data analysis, and drafted the manuscript. KC carried out surgical procedures, TTC staining, in vivo measurements, and analysed rat data. SL performed surgical procedures, in vivo measurements and data analysis in mice. DY/DH/ML participated in study design, coordination and sourced funding. Additionally, ML critically revised the intellectual content of the manuscript. All authors read and approved the final draft.

## References

[B1] Epstein FH MR in mouse models of cardiac disease Nmr in Biomedicine 2007 20 238 255 10.1002/nbm.1152 17451182

[B2] Hoit BD New approaches to phenotypic analysis in adult mice Journal of Molecular and Cellular Cardiology 2001 33 27 35 10.1006/jmcc.2000.1294 11133220

[B3] Price AN Cheung KK Cleary JO Campbell AE Riegler J Lythgoe MF Cardiovascular magnetic resonance imaging in experimental models Open Cardiovasc Med J 2010 4 278 292 10.2174/1874192401004010278 21331311 PMC3040459

[B4] Schneider JE Cassidy PJ Lygate C Tyler DJ Wiesmann F Grieve SM Fast, high-resolution in vivo cine magnetic resonance imaging in normal and failing mouse hearts on a vertical 11.7 T system Journal of Magnetic Resonance Imaging 2003 18 691 701 10.1002/jmri.10411 14635154

[B5] Schneider JE Lanz T Barnes H Medway D Stork LA Lygate CA Ultra-fast and accurate assessment of cardiac function in rats using accelerated MRI at 9.4 Tesla Magnetic Resonance in Medicine 2008 59 636 641 10.1002/mrm.21491 18306411

[B6] Vallee JP Ivancevic MK Nguyen D Morel DR Jaconi M Current status of cardiac MRI in small animals Magnetic Resonance Materials in Physics Biology and Medicine 2004 17 149 156 10.1007/s10334-004-0066-4 15605278

[B7] Kim RJ Wu E Rafael A Chen EL Parker MA Simonetti O The use of contrast-enhanced magnetic resonance imaging to identify reversible myocardial dysfunction N Engl J Med 2000 343 1445 1453 10.1056/NEJM200011163432003 11078769

[B8] Kim RJ Fieno DS Parrish TB Harris K Chen EL Simonetti O Relationship of MRI delayed contrast enhancement to irreversible injury, infarct age, and contractile function Circulation 1999 100 1992 2002 10556226 10.1161/01.cir.100.19.1992

[B9] Simonetti OP Kim RJ Fieno DS Hillenbrand HB Wu E Bundy JM An improved MR imaging technique for the visualization of myocardial infarction Radiology 2001 218 215 223 11152805 10.1148/radiology.218.1.r01ja50215

[B10] Yang Z Berr SS Gilson WD Toufektsian MC French BA Simultaneous evaluation of infarct size and cardiac function in intact mice by contrast-enhanced cardiac magnetic resonance imaging reveals contractile dysfunction in noninfarcted regions early after myocardial infarction Circulation 2004 109 1161 1167 10.1161/01.CIR.0000118495.88442.32 14967719

[B11] Epstein FH Yang ZQ Gilson WD Berr SS Kramer CM French BA MR tagging early after myocardial infarction in mice demonstrates contractile dysfunction in adjacent and remote regions Magnetic Resonance in Medicine 2002 48 399 403 10.1002/mrm.10210 12210951

[B12] Bohl S Lygate CA Barnes H Medway D Stork LA Schulz-Menger J Advanced methods for quantification of infarct size in mice using three-dimensional high-field late gadolinium enhancement MRI Am J Physiol Heart Circ Physiol 2009 296 H1200 H1208 10.1152/ajpheart.01294.2008 19218501 PMC2670705

[B13] Chapon C Herlihy AH Bhakoo KK Assessment of myocardial infarction in mice by late gadolinium enhancement MR imaging using an inversion recovery pulse sequence at 9.4T J Cardiovasc Magn Reson 2008 10 6 10.1186/1532-429X-10-6 18272007 PMC2244610

[B14] Ojha N Roy S Radtke J Simonetti O Gnyawali S Zweier JL Characterization of the structural and functional changes in the myocardium following focal ischemia-reperfusion injury Am J Physiol Heart Circ Physiol 2008 294 H2435 H2443 10.1152/ajpheart.01190.2007 18375718 PMC4772869

[B15] Thomas D Dumont C Pickup S Misselwitz B Zhou R Horowitz J T1-weighted cine FLASH is superior to IR imaging of post-infarction myocardial viability at 4.7T J Cardiovasc Magn Reson 2006 8 345 352 10.1080/10976640500451986 16669177 PMC2581493

[B16] Protti A Sirker A Shah AM Botnar R Late gadolinium enhancement of acute myocardial infarction in mice at 7T: cine-FLASH versus inversion recovery J Magn Reson Imaging 2010 32 878 886 10.1002/jmri.22325 20882618

[B17] Graumann R Deimling M Heilmann T Oppelt A A new method for fast and precise T_1 _determination Proceedings of the Society of Magnetic Resonance in Medicine 1986 1986 922 923

[B18] Look DC Locker DR Time saving in measurement of NMR and EPR relaxation times Review of Scientific Instruments 1970 41 250 251 10.1063/1.1684482

[B19] Brix G Schad LR Deimling M Lorenz WJ Fast and Precise T1 Imaging Using A Tomrop Sequence Magnetic Resonance Imaging 1990 8 351 356 10.1016/0730-725X(90)90041-Y 2392022

[B20] Schneider JE Wiesmann F Lygate CA Neubauer S How to perform an accurate assessment of cardiac function in mice using high-resolution magnetic resonance imaging Journal of Cardiovascular Magnetic Resonance 2006 8 693 701 10.1080/10976640600723664 16891228

[B21] Kellman P McVeigh ER Image reconstruction in SNR units: a general method for SNR measurement Magn Reson Med 2005 54 1439 1447 10.1002/mrm.20713 16261576 PMC2570032

[B22] Heiberg E Wigström L Carlsson M Bolger AF Karlsson M Time Resolved Three-dimensional Automated Segmentation of the Left Ventricle Proceedings of IEEE Computers in Cardiology 2005 32 599 602

[B23] Riegler J Cheung KK Man YF Cleary JO Price AN Lythgoe MF Comparison of segmentation methods for MRI measurement of cardiac function in rats J Magn Reson Imaging 2010 32 869 877 10.1002/jmri.22305 20882617

[B24] Jivan A Horsfield MA Moody AR Cherryman GR Dynamic T-1 measurement using snapshot-FLASH MRI Journal of Magnetic Resonance 1997 127 65 72 10.1006/jmre.1997.1177 9245631

[B25] Oshinski JN Yang ZQ Jones JR Mata JF French BA Imaging time after Gd-DTPA injection is critical in using delayed enhancement to determine infarct size accurately with magnetic resonance imaging Circulation 2001 104 2838 2842 10.1161/hc4801.100351 11733404

[B26] Judd RM Kim RJ Imaging time after Gd-DTPA injection is critical in using delayed enhancement to determine infarct size accurately with magnetic resonance imaging Circulation 2002 106 E6 10.1161/01.CIR.0000019903.37922.9C 12105174

